# Primary Stroke Center Concept: Strengths and Limitations

**DOI:** 10.3389/fneur.2012.00108

**Published:** 2012-07-18

**Authors:** Farrukh Salim Chaudhry, Michael J. Schneck, J. Warady, J. Platakis, Sarkis Gibran Morales-Vidal, Jose Biller, M. Flaster

**Affiliations:** ^1^Neurology, Loyola University Medical CenterMaywood, IL, USA

The American Heart Association estimates that, as of calendar year 2010, approximately 795,000 people in the USA will have experienced a new or recurrent stroke, out of which 610,000 were new attacks (Heart Disease and Stroke Statistics – 2010 Update, 2010). Stroke is the third leading cause of death in United States and a major cause of disability. There are almost three million survivors of stroke, most of whom are disabled with a societal cost of approximately $30 billion (US dollars; Heart Disease and Stroke Statistics – 2010 Update, 2010). Over the last two decades, there have been major advances have been made in the early diagnosis of stroke and timely treatment. In 1996, the US Food and Drug Administration (USFDA) approved intravenous tissue plasminogen activator (IV tPA) for the treatment of acute ischemic stroke within 3 h of onset of symptoms (The National Institute of neurological Disorders and Stroke rt-PA Stroke Study Group, 1995). Despite the approval, only 1.8–4.1% of patients with acute ischemic stroke received IV tPA (Katzan et al., 2000). The failure to administer tPA in a timely fashion was evident from a paper, in calendar year 2000, showing that only 34% of academic hospitals had defined stroke protocols and only 18% had rapid identification methods for patients presenting with acute stroke symptoms (Johnson et al., 2001). A subsequent assessment of statewide acute stroke care in Illinois demonstrated that although 93.2% of residents in Illinois lived in a county with at least one acute care facility with tPA treatment protocol, there were many acute care receiving facilities outside of Greater Chicago Metropolitan Area which did not have a neurologist or a neurosurgeon available (Ruland et al., 2002).

In response, a group of medical organizations joined forces in an effort to improve this situation. The result was a project called “Brain Attack: A Body of Knowledge, A Coalition of Support.” In 2000, the Brain Attack Coalition (BAC) published their initial recommendations for the establishment of primary stroke centers (PSC) to improve the care of patients with stroke (Alberts et al., 2000). These PSCs would have designated acute stroke teams, stroke units, written care protocols, and an integrated emergency response system along with quick access to ancillary investigations including laboratory studies and computerized tomography (CT; Alberts et al., 2000). There was also a proposal for establishment of two levels of care for acute stroke patients based on the trauma level system of care (Schneck, 1998). The PSC would stabilize and treats acute stroke patient and provide them with initial care including intravenous thrombolytic therapy utilizing tPA, as well as other acute therapies such as stabilization of vital functions, provision of neuroimaging procedures, and management of intracranial pressure (Alberts et al., 2000). However, patients with more difficult to treat or more complex strokes with severe deficits and multi-organ involvement would require more resources and a higher intensity of care than is offered by the PSC. In these situations, PSC could transfer patients to a comprehensive stroke center (CSC; Alberts et al., 2005). The CSC would treat acute stroke patient at all needed levels of care and offer specialized care and interventions for highly technical procedures such as intra-arterial thrombolysis, neurosurgical, and neurointensive care requiring specialized infrastructure and personnel (Alberts et al., 2005).

After an extensive review of published literature concerning the establishment of medical centers, with a focus on stroke and trauma centers, the BAC identified 11 major elements to be incorporated in a PSC, which are highlighted in Table 1 (Alberts et al., 2000). The BAC endorsed formal certification programs such as the Joint Commission Disease-Specific Certification process. By, January 2006, there were 174 PSCs certified by the Joint Commission on Accreditation of Healthcare Organizations (Joint Commission) and by October 2009 there were over 600 certified centers^1^. The Joint Commission and National Quality Forum endorsed 10 performance measures listed in Table 2, for programs seeking certification as stroke centers. In 2010, the Joint commission eliminated the requirement for documentation of dysphagia screening while making the smoking cessation requirement universal. The ostensible reason for eliminating dysphagia screening was because of a lack of consensus on screening methods for dysphagia (see text footnote 1). US Centers for Medicare and Medicaid Services (CMS) is adopting these eight measures and considering whether to require them for future reporting possibly starting in 2012 in all hospitals that care for stroke^2^. In addition, starting in 2010 hospitals submitting Medicare claims must inform CMS whether they participate in a stroke care registry (see text footnote 2).

**Table 1:** **Major elements of a primary stroke center or PSC (Alberts et al., 2000)**.

Acute stroke teams
Written care protocol
Emergency medical services
Emergency department
Stroke unit (if patients are admitted)
Neurosurgical services
Commitment and support of a medical organization including a stroke center director
Neuroimaging services
Laboratory services
Outcome and quality improvement activities
Continuing medical education

**Table 2:** **JCAHO standardize stroke performance measures (http://www.jointcommission.org/AboutUs/Fact_Sheets/psc_certification.htm)**.

Deep vein thrombosis prophylaxis
Discharged on antithrombotic therapy
Anticoagulation therapy for atrial fibrillation
Thrombolytic therapy administered to eligible patients
Antithrombotic therapy by end of hospital day 2
Discharged on lipid lowering medication if warranted (LDL > 100, not measured, or on medication prior to admission)
Dysphagia screening*
Stroke education
Smoking cessation/advice/counseling*
Assessed for rehabilitation

*Retired effective January 2010.

While the PSC requirements have been well-defined, there are as yet no formal networks of care based on the CSC concept. The BAC suggested the following elements be present for CSCs (Alberts et al., 2005):

1. Health care personnel with specific expertise in a number of disciplines, including neurosurgery and vascular neurology (but not neurointensivists).
2. Advanced neuroimaging capabilities such as MRI and various types of cerebral angiography.
3. Surgical and endovascular capabilities, including clipping and coiling of intracranial aneurysms, carotid endarterectomy, and intra-arterial thrombolytic therapy.
4. Other specific infrastructure and programmatic elements such as an intensive care unit and a stroke registry.

The impact of PSC designation on improving stroke care has been positively described in multiple reports. Gropen et al. (2006) compared the quality of care provided by PSC in acute stroke settings in New York state designated stroke centers vs. non-designated hospitals. In this study, the authors examined the outcome of stroke systems that linked early stroke recognition and transport to stroke centers to delivery of care. They concluded that ischemic stroke patients evaluated in a designated facility were seen by physicians twice as quickly once they reached the hospital, head CTs were performed twice as rapidly for appropriate tPA candidates, and use of tPA more than doubled without any increase in protocol violations or complications. The number of admissions to stroke units was likewise higher in designated facilities, which is significant given the lower mortality and better functional recovery associated with these specialized units (Gropen et al., 2006).

Similar results were reported in European stroke systems. Meretoja et al. (2010), looked at effectiveness of all primary and CSCs in Finland that fulfill standardized BAC criteria by reviewing data from all patients with ischemic stroke from 1999 to 2006. These observations concluded that care in stroke centers was associated with lower 1-year case-fatality and reduced post-acute institutional care compared with general hospitals. The number-needed-to-treat to prevent one death or institutional care at 1 year was 29 for CSC equivalent hospitals and 40 for PSC equivalent hospitals compared to general hospitals. Patients treated in stroke centers had lower mortality during the entire follow-up of up to 9 years and their median survival was increased by 1 year (Meretoja et al., 2010).

The impact of establishing designated stroke centers has been often assessed by the use of thrombolytic therapy. In the NINDS suburban hospital stroke center experience in Bethesda, the rates if tPA administration at a community hospital before and after stroke center designation were assessed (Lattimore et al., 2003). Prior to the designation, the hospital lacked an acute stroke response team, 24 months after creation of an acute stroke team there was a sevenfold increase in tPA administration (1.5–10.5%). In addition, there was a decrease in the time from patient arrival at hospital to paging of the stroke team, time of stroke team page to team arrival, and time to screening brain scan over the 24-month period (Lattimore et al., 2003). Wojner-Alexandrov et al. (2005), reported similar increases in use of tPA after BAC and American Stroke Association (ASA) guidelines were implemented over 1 year in two Houston Texas hospitals which had specialized stroke programs. Interestingly, these pre-existing stroke centers had significantly lower emergency department arrival to CT interpretation times when compared to those of newly established stroke centers (Wojner-Alexandrov et al., 2005).

Although the increased use of tPA has been associated with stroke center designation in both primary and comprehensive facilities, Douglas et al. (2005) reported that of the 11 elements recommended by BAC, only seven were related to increased tPA use. The study concluded that variables associated with higher tPA use were related to reducing delays in treatment, including emergency medical services, organized emergency departments, rapid neuroimaging, and an acute stroke team. Also, continuing medical education was found to increase tPA administration, by probably decreasing the delay to treatment. Interestingly, the study was unable to demonstrate that any of the 11 elements were significantly related to decreased inpatient mortality or increased frequency of discharge home (Douglas et al., 2005). Furthermore, there was a lack of correlation between stroke units and decreased in-hospital mortality, a conclusion seemingly at odds with previous reports that clearly demonstrated that stroke unit care is associated with reduced mortality and better functional outcome (Stroke Unit Trialists’ Collaboration, 2007).

One important requirement for establishment of a PSC is community education and pre-hospital screening of stroke patients. In 2005, the Behavioral Risk Factor Surveillance System (BRFSS) reported that out of those screened, only 38.1% were aware of five stroke warning symptoms or signs and would first call “911” if they thought someone is having a stroke or a heart attack [Centers for Disease Control and Prevention (CDC), 2008]. About 41.3% of these respondents were white, 29.5% African-Americans, and 26.8% were Hispanic [Centers for Disease Control and Prevention (CDC), 2008]. Similarly, other studies including random telephonic surveys concluded that there is insufficient awareness in community about risk factors, warning signs, and prevention strategies of stroke. Wall et al. (2008) with the Massachusetts Heart Disease and Stroke Prevention Program report that measures such as Stroke Heroes Act FAST media and public awareness campaign is extremely important because stroke outcome may not improved unless people recognize the warning signs of stroke and activate the system by calling 911, regardless of attempts to improve EMS and hospital emergency department systems.

Education for health care providers is also lacking. For example, a survey of 308 internal medicine residency programs showed that only 46% required rotation in neurology; in contrast 97% required the study of cardiology (Maron et al., 2005). The report concluded that under-representation of neurology training amongst internists may lead to under-recognition of stroke signs and symptoms and may effect stroke outcome. There is also ongoing resistance to thrombolytic therapy among a sub-segment of emergency room physicians which may partly reflect lack of education but also lack of organized systems of care. In a 2005 report, 40% of emergency physicians were unlikely to give tPA to stroke patients because of fear of complications (Brown et al., 2005). With the development of stroke systems of care, however, emergency physicians have gradually become more accepting of stroke thrombolysis within the context of formal guidelines and PSC systems.

The stroke center concept remains limited, however, in its primary focus on thrombolytic therapy for acute ischemic stroke. Even at the “best” centers, however, only 15–20% of patients will be eligible for thrombolytic therapy (Katzan et al., 2000; Johnson et al., 2001; Wojner-Alexandrov et al., 2005). An equally important focus of stroke centers must be on non-tPA stroke issues. For example, a lack of standardized measures for the assessment of PSC and CSC management of hemorrhagic stroke is a glaring deficit, especially as patients with hemorrhagic stroke (both intracerebral and subarachnoid hemorrhage) comprise roughly 20% of all stroke patients (Broderick et al., 1999; Heart Disease and Stroke Statistics – 2010 Update, 2010). Additionally, there is no reference to carotid artery disease in any of the stroke center requirements despite a robust set of evidence based data guiding the treatment of both symptomatic and asymptomatic carotid disease (Chaturvedi et al., 2005). Furthermore, an increasing important component of stroke center therapy related to endovascular interventions is not currently addressed in the PSC process. This becomes more important as the collection of evidence based randomized data to support endovascular treatments (IA therapies) accumulates. At present, patients who are ineligible for intravenous tPA who might still be eligible for IA therapies may not be taken, via bypass mechanisms even if available, to the nearest IA capable center but rather, at best, only to the nearest PSC.

Finally, while the PSC process has clearly improved the level of care delivered to stroke patients, adherence to recommended national quality guidelines was also associated with improvements in a number of the composite measures when an organized system based on the Get-with-the Guidelines database was implemented. Improvements were seen in all seven individual measures quality improvement measures (Schwamm et al., 2009). The documentation requirements, however, are often burdensome, and a “one-size-fit all” template approach may result in inappropriate patient evaluations. Thus, while the PSC concept has improved the overall quality of care, the lack of organized stroke systems, with appropriate pre-hospital triage, lack of diversion of patients to appropriate levels of care, shortage of stroke expertise, and problems with extension of acute care to other types and facets of complex stroke care are ongoing challenges that must be addressed if the stroke center concept is to prove workable and more effective on a nationwide scale.

## Footnotes

^1^http://www.jointcommission.org/AboutUs/Fact_Sheets/psc_certification.htm

^2^http://newsroom.heart.org/pr/aha/805.aspx
